# Psilocybin-assisted physiotherapy for refractory motor functional neurological disorder: protocol for a randomised dose-comparison pilot study

**DOI:** 10.1017/neu.2025.10042

**Published:** 2025-11-04

**Authors:** Chiranth Bhagavan, Alexander Bryson, Olivia Carter, Glenn Nielsen, David Berlowitz, Sara Issak, Sabine Braat, Sophie Zaloumis, Zachary Attard, Dina Eleftheriadis, Georgina Oliver, Deanne Mayne, Greg Roebuck, James Rucker, Matthew Butler, Richard Kanaan

**Affiliations:** 1 Department of Psychiatry, University of Melbourne, Austin Health, Heidelberg, VIC, Australia; 2 Florey Institute of Neuroscience & Mental Health, Parkville, VIC, Australia; 3 Department of Neurology, Melbourne Health, Parkville, VIC, Australia; 4 Department of Neurology, Eastern Health, Box Hill, VIC, Australia; 5 Melbourne School of Psychological Sciences, University of Melbourne, Parkville, VIC, Australia; 6 Neurosciences and Cell Biology Research Institute, St George’s University of London, London, UK; 7 Institute for Breathing and Sleep, Austin Health, Heidelberg, VIC, Australia; 8 Department of Physiotherapy, Melbourne School of Health Sciences, University of Melbourne, Carlton, VIC, Australia; 9 Department of Physiotherapy, Epworth Healthcare, Camberwell, VIC, Australia; 10 Centre for Epidemiology and Biostatistics, Melbourne School of Population and Global Health, University of Melbourne, Melbourne, VIC, Australia; 11 MISCH (Methods and Implementation Support for Clinical Health) Research Hub, Faculty of Medicine, Dentistry and Health Sciences, University of Melbourne, Melbourne, VIC, Australia; 12 FND Hope International, Salmon, ID, USA; 13 Phoenix Australia, Centre for Posttraumatic Mental Health, Department of Psychiatry, University of Melbourne, Carlton, VIC, Australia; 14 The Institute for Mental and Physical Health and Clinical Translation (IMPACT), School of Medicine, Deakin University and Barwon Health, Geelong, VIC, Australia; 15 Department of Psychological Medicine, Institute of Psychiatry, Psychology & Neuroscience, King’s College London, London, UK; 16 South London & Maudsley NHS Foundation Trust, Bethlem Royal Hospital, Beckenham, UK

**Keywords:** Psychiatry, neuropsychiatry, psilocybin, conversion disorder, physiotherapy

## Abstract

**Background:**

Motor functional neurological disorder (FND) is a common illness associated with significant functional impairment. There are no effective pharmacotherapies, and despite the early promise of physiotherapy studies, many suffer disabling symptoms in the long term. There is a theoretical rationale for combining psychedelics with physiotherapy; however, the potential benefit of this approach and optimal treatment model remains unexplored. Here, we present the protocol for the first study investigating the tolerability, feasibility, and potential efficacy of two distinct treatment regimens of psilocybin-assisted physiotherapy for refractory motor FND: a moderate dose that incorporates movement tasks during the acute drug effects versus a standard dose alone.

**Methods:**

Twenty-four participants with refractory motor FND will be randomised in a 1:1 ratio to either (1) psilocybin 15 mg, with movement tasks conducted during the acute drug effects, or (2) psilocybin 25 mg alone. All participants will receive two sessions of FND-specific physiotherapy pre-dosing, six sessions of physiotherapy post-dosing, and undergo follow-up visits one week and four weeks following their final physiotherapy session. A battery of outcome measures will be completed as scheduled, assessing tolerability, feasibility, motor FND symptom severity, psychiatric and physical symptoms, quality of life, treatment expectations, intensity of the acute drug effects, personality, motor function, force-matching performance, resting-state and task-based brain imaging, and subjective experiences of the study treatment.

**Discussion:**

These findings will assist the design of an adequately powered randomised controlled trial in this cohort. The findings may also inform the feasibility of psychedelic treatment in related functional and neuropsychiatric disorders.

## Graphical abstract

**Figure:**
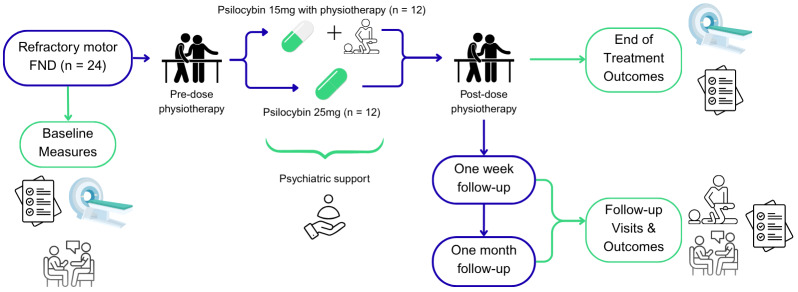

## Summations

- Motor functional neurological disorder (FND) poses a substantial clinical burden and has no effective pharmacotherapies. Building upon the encouraging treatment potential of psychedelics in neuropsychiatric disorders, this protocol outlines the first study investigating psilocybin-assisted physiotherapy for refractory motor FND.
- The study will compare a moderate dose of psilocybin that incorporates movement tasks during the acute drug effects versus a conventional, standard-dose approach. Both treatment groups will receive a course of FND-specific physiotherapy pre- and post-dosing, accompanied by psychiatric support. A battery of measures will be assessed at baseline and up to four weeks post-treatment.
- The findings will inform an adequately powered randomised controlled trial in this cohort and provide insights into the feasibility of psychedelic treatment in other functional and neuropsychiatric disorders.

## Considerations

- Novel therapies for motor functional neurological disorder (FND) are required because existing treatments are insufficient. Supported by a theoretical basis, psychedelic-assisted physiotherapy in motor FND warrants further investigation.
- Testing psychedelics with physiotherapy will also provide novel insights into the potential for psychedelic-assisted therapy to target broader physiological and sensorimotor mechanisms implicated in other functional and neuropsychiatric disorders.
- Given the nascency of this area, this pilot study will prioritise safety and feasibility, with any observed benefits limited by the study’s open-label design and small sample size. As the field evolves, randomised controlled studies with adequate power and long-term outcomes will contribute to understanding the translational potential of this treatment.

## Highlights

- Motor functional neurological disorder is a common illness. Many individuals do not respond to current treatments and experience disabling symptoms in the long term.
- Building on the encouraging treatment potential of psychedelics in neuropsychiatric disorders and their hypothesised mechanism of action, this protocol details a study comparing the feasibility and potential efficacy of two distinct treatment regimens of psilocybin combined with physiotherapy.
- The findings will inform a follow-up, randomised controlled trial in refractory motor functional neurological disorder.

## Introduction

### Background and rationale

Functional neurological disorder (FND) presents with neurological symptoms that are considered incompatible with other neurological conditions (Hallett *et al*., 2022). Motor FND refers to the presence of functional motor symptoms, such as weakness, tremor, or abnormal gait. It is a common illness, with estimates of incidence of functional motor symptoms at approximately 4–5/100’000 population per year (Binzer *et al*., 1997; Stone *et al*., 2010). These are almost certainly underestimates, as many remain undiagnosed for years (Crimlisk *et al*., 2000), and presentations have likely increased since the coronavirus disease 2019 pandemic (Hull *et al*., 2021). Many individuals continue to experience disabling symptoms in the long term, as demonstrated by a systematic review which found that 39% of individuals with functional motor symptoms remained the same or worse at follow-up (Gelauff *et al*., 2014).

Available treatment options in motor FND are limited. Pharmacotherapy is not indicated in its direct treatment (Aybek & Perez, 2022), and trials of psychotherapy remain inconclusive (Hinson *et al*., 2006; Kompoliti *et al*., 2014; Dallocchio *et al*., 2016). Preliminary evidence from pilot studies demonstrated symptom improvement with psychologically informed, FND-specific physiotherapy (Czarnecki *et al*., 2012; Jordbru *et al*., 2014; Nielsen *et al.,* 2017b). However, a recently published phase 3 randomised controlled trial (RCT) of FND-specific physiotherapy for motor FND demonstrated mixed findings (Nielsen *et al*., 2024). Although a significant difference in the primary outcome measure was not observed, the physical functioning domain of the participant-reported 36-Item Short Form Survey (SF-36) indicated that 59% of the specialist physiotherapy group rated their symptoms as improved compared to 39% of the standard physiotherapy group. This suggests that while a proportion of individuals may benefit from physiotherapy alone, alternative approaches are necessary for those who are refractory to treatment.

While pathophysiological models of motor FND remain an evolving field, several proposed mechanisms have been consolidated into a Bayesian approach based upon the dynamic relationship between sensory input and neural representations of this information (Friston, 2010; Edwards *et al*., 2012). This model posits that in motor FND, top-down somatic self-representations, or priors, may become overly precise through factors such as abnormal emotion processing, self-directed attention, and interoceptive dysfunction. In the context of motor control, this generates a disconnect between conscious experience and sensorimotor function, resulting in motor symptoms. Furthermore, these symptoms may become entrenched when interpreted as illness, thus reinforcing aberrant self-representations.

Based on this model, there is emerging interest in the application of classic psychedelics, such as psilocybin, for motor FND (Bryson *et al*., 2017). Psychedelics exert multifaceted effects upon the brain, including synaptogenesis and dendritic arborisation (Ly *et al*., 2018); altered connectivity across macroscopic cortical networks (Madsen *et al*., 2021); and both acute changes in perception and conscious state and longer-term changes in beliefs and behaviours (Studerus *et al*., 2011). A synthesis of these effects suggests that psychedelics can promote neuroplasticity and relax overly precise priors while increasing the brain’s sensitivity to sensory input (Carhart-Harris & Friston, 2019). Indeed, these changes are hypothesised to underlie their therapeutic effects in several neuropsychiatric disorders, including major depressive disorder (MDD), illness-related anxiety, obsessive compulsive disorder, and substance use disorder (Andersen *et al*., 2021).

The impact of psychedelics upon brain function suggests a promising role for psychedelic-assisted physiotherapy in motor FND. By relaxing somatic priors, sensorimotor processes may become freed from maladaptive top-down influences and more receptive to sensory input, such as proprioceptive feedback during movement retraining, thus enhancing the therapeutic potential of physiotherapy (Bryson *et al*., 2017). Although an encouraging exploration of psychedelics in FND was underway before the prohibition of these agents, the studies were limited by poor quality, and psychedelic treatment by itself was not universally effective (Butler *et al*., 2020). This may suggest a potential role for augmenting psychedelics with modern physiotherapy approaches.

Combining psychedelic treatment with physiotherapy for motor FND raises questions regarding the most appropriate treatment regimen. In previous studies of psychedelics in FND, a ‘psycholytic’ regimen was used in most cases, which involves low-to-moderate doses (typically 3 to 15 mg of psilocybin) combined with psychotherapy during the dosing session (Passie *et al*., 2022). An analogous approach could be used in psychedelic-assisted physiotherapy, whereby the participant engages in movement tasks during the drug effects to take advantage of acute changes in brain dynamics and neuroplasticity following psychedelic administration (Carhart-Harris & Friston, 2019; Berkovitch *et al*., 2025; Weiss *et al*., 2025). We recently completed a dose-finding pilot study of movement tasks undertaken during the acute effects of low-to-moderate psilocybin doses in healthy participants – the first study of its kind to assess the impact of psilocybin on motor function (Bhagavan *et al*., 2024) – which demonstrated the feasibility of engaging in movement tasks during the acute effects of psilocybin up to 15 mg. However, whether it is also feasible to administer physiotherapy during the acute psychedelic effects in motor FND remains uncertain.

In contrast, modern studies have predominantly adopted a ‘psychedelic’-assisted therapy (PAT) framework involving higher doses (typically 25 mg of psilocybin) and more profound psychoactive effects (Barber and Aaronson, 2022; Passie *et al*., 2022). In studies of depression and anxiety, psychotherapy is also provided in the weeks following dosing to leverage a persistent ‘window’ of neuroplasticity, the duration of which may correlate with the intensity of the psychedelic experience (Watts and Luoma, 2020; Lepow *et al*., 2021; Nardou *et al*., 2023). Given the potential practical challenges of administering physiotherapy during acute psychedelic effects, this presents an alternative treatment model in motor FND: a standard ‘psychedelic’ dose alone, followed by physiotherapy in the subsequent weeks. However, whether this sustained window of neuroplasticity fosters movement retraining is also unknown. Ultimately, the optimal psilocybin dose and application of adjunctive physiotherapy for motor FND are unclear and in need of further exploration.

This protocol, therefore, details an individually randomised parallel-group pilot study assessing the tolerability, feasibility, and potential efficacy of psilocybin-assisted physiotherapy in participants with refractory motor FND. This study will compare two distinct psilocybin-assisted treatment paradigms: 1) a moderate ‘psycholytic’ dose (15 mg) that incorporates movement tasks during the acute drug effects, and 2) a standard ‘psychedelic’ dose (25 mg), integrated within a course of FND-specific physiotherapy for both treatment groups.

### Objectives

#### Hypotheses

1. Both standard- and moderate-dose psilocybin will be well-tolerated in participants with refractory motor FND.
2. It is feasible for participants with refractory motor FND to perform a series of movement tasks during the acute effects of moderate-dose psilocybin.
3. Psilocybin-assisted physiotherapy can improve motor symptoms and disability in participants with refractory motor FND compared to the participants’ baseline assessment.
4. There is a difference in tolerability and symptom improvement between moderate- and standard-dose psilocybin-assisted physiotherapy in participants with refractory motor FND.

#### Primary aims

- To assess the tolerability of psilocybin-assisted physiotherapy in participants with refractory motor FND and compare between treatment groups, as measured by vital signs and adverse event (AE) reporting.
- To assess the feasibility of completing movement tasks during the acute effects of moderate-dose psilocybin in participants with refractory motor FND.
- To assess the effect of psilocybin-assisted physiotherapy in participants with refractory motor FND on within- and between-group changes in:
  - Clinician-rated changes in motor FND symptoms, using the Simplified Functional Movement Disorder Rating Scale (S-FMDRS).
  - Participant-reported changes in motor FND severity and improvement, using the Patient Global Impression of Severity (PGI-S) and Improvement (PGI-I).

#### Secondary aims

- To assess the effect of psilocybin-assisted physiotherapy in participants with refractory motor FND on within- and between-group changes in:
  1. Motor FND symptom severity and improvement, using the Clinical Global Impression of Severity (CGI-S) and Improvement (CGI-I).
  2. Motor FND symptom severity, using the S-FMDRS, graded through video assessment by an independent observer.
  3. Depressive symptoms, using the Patient Health Questionnaire-9 (PHQ-9).
  4. Anxiety symptoms, using the Generalised Anxiety Disorder-7 (GAD-7).
  5. Somatic symptoms, using the Patient Health Questionnaire-15 (PHQ-15).
  6. Health-related quality of life, using the SF-36.
- To assess the impact of pre-treatment expectations on outcomes in participants with refractory motor FND and compare between groups, using the Stanford Expectations of Treatments Scale (SETS).
- To assess the impact of psilocybin on altered conscious states and ego-dissolution in participants with refractory motor FND and compare between groups, using the 5-Dimensional Altered States of Consciousness (5D-ASC) and Ego-Dissolution Inventory (EDI).
- To determine the utility of these outcome measures for an adequately powered RCT of psilocybin-assisted physiotherapy in motor FND.

#### Exploratory aims

- To explore any acute benefits of performing movement tasks during the acute effects of moderate-dose psilocybin in participants with refractory motor FND.
- To explore the impact of psilocybin-assisted physiotherapy in participants with refractory motor FND on within- and between-group changes in:
  - Motor function, using the De Morton Mobility Index (DEMMI), Functional Movement Exploration (FME), Action Research Arm Test (ARAT), Box and Block Test (BBT) – original and modified versions – and video footage.
  - Sensorimotor function, using the force-matching task.
  - Resting-state and task-based measures of functional brain activity, using functional magnetic resonance imaging (fMRI).
  - Personality traits, using the Big Five Inventory-2 (BFI-2).
  - Experiences of the study treatment, using a study treatment questionnaire.
  - Qualitative effects through face-to-face interviews.

### Trial design

This is a two-arm, individually randomised parallel-group study in 24 participants with refractory motor FND who will be randomly assigned, in a 1:1 ratio, to one of two treatment groups:

- Psilocybin 15 mg with movement tasks during the acute drug effects (moderate-dose arm), or
- Psilocybin 25 mg without movement tasks during dosing (standard-dose arm).

**Figure 1.**
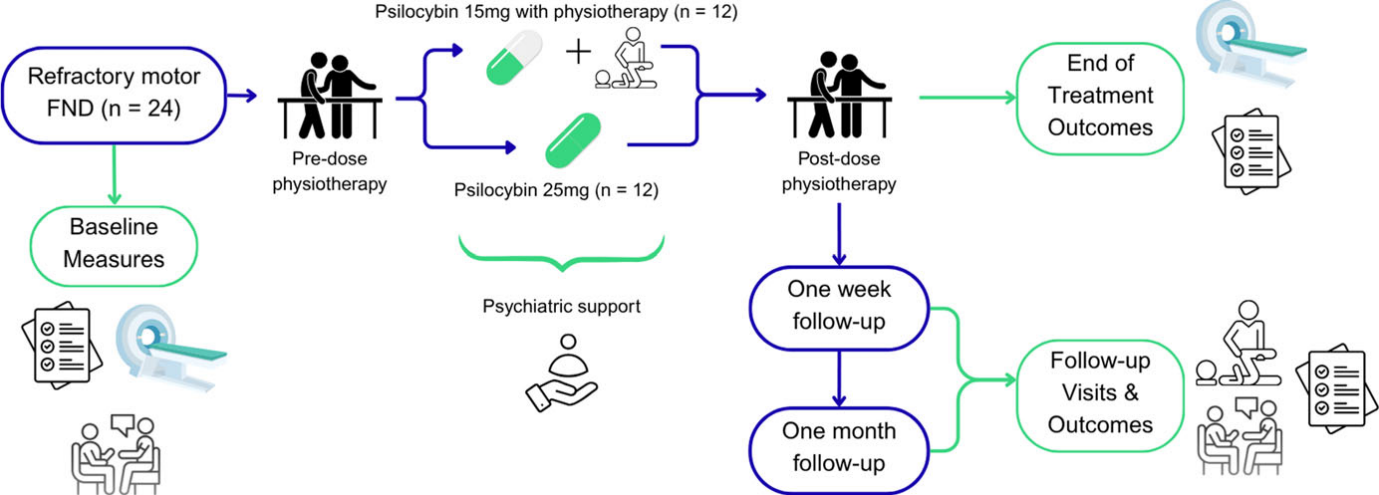
Study Design. Abbreviation: FND, Functional Neurological Disorder.

All participants will receive specialist, FND-specific physiotherapy, including two sessions pre- and six sessions post-dosing. Physiotherapy follow-up visits will occur one week and four weeks after completing their treatment. Psychiatric oversight will be provided, including a preparation session before their psilocybin dose, supervision during the dosing session, and follow-up sessions one week and four weeks after completing their physiotherapy treatment. An overview of this design is outlined in Fig. 1. Statisticians and an independent assessor of symptom severity will remain blinded to treatment allocation.

### Sample size

A sample size of 12 has been chosen because, assuming an 80% chance of a participant successfully completing physiotherapy during acute dosing, this provides over a 90% chance of enrolling at least one participant who is unable to complete the intervention. We consider a completion rate of at least 80% to be tolerable, and so a sample size of 12 is likely sufficient to screen for a poorly tolerated intervention. Participants who withdraw before psilocybin dosing will be replaced.

## Methods: participants, interventions, and outcomes

### Study setting

All study visits will occur at Austin Health, a public tertiary teaching hospital in Melbourne, Australia. fMRI scans will occur at the Melbourne Brain Centre, a research centre at the hospital. The dosing session will occur within a dedicated room providing a comfortable, monitored, clinical setting with access to temperature control, blankets, headphones, and music. Physiotherapy treatment sessions and follow-up visits will occur within the same room. Psychiatry follow-up visits and remaining scheduled outcome measures will occur within the study psychiatrist’s office.

### Eligibility criteria

#### Study team eligibility

The trial physiotherapists will be registered physiotherapists trained in the Physio4FMD specialist physiotherapy intervention (Nielsen & Holt, 2024) and movement tasks administered. The psychiatrists will be registered psychiatrists with experience working with psychedelic medicines.

### Recruitment

The treating psychiatrist at Austin Health’s Functional Neurology Clinic will discuss the study with potentially eligible participants. The study team will also distribute a referral flyer to clinicians in Australia working with FND. Interested individuals will then be referred to the study by their treating practitioner and provided with the participant information sheet and consent form (PICF), which contains detailed study information and has been approved by the Austin Health Human Research Ethics Committee (AHHREC).

### Consent and screening

Referred participants will be invited to a screening visit with the study psychiatrist, who will undertake informed consent. Information about the study procedures, potential benefits, and risks will be provided in a clear, balanced, and neutral manner. Informed consent will be obtained for the use of de-identified data for both this study and secondary uses, including informing future related projects and safety data to be shared with the Usona Institute.

Those willing to proceed with the study will provide a dated signature on the PICF. The study psychiatrist will then conduct a medical and psychiatric history, physical examination, vital signs, and electrocardiogram to assess eligibility.

### Assignment of interventions

#### Allocation sequence

Participants will be randomised in a 1:1 ratio into one of the two treatment groups until 12 participants in each group have been allocated. The randomisation list will be computer-generated by an independent statistician of the University of Melbourne, using random block sizes, and uploaded as a randomisation module into Research Electronic Data Capture (REDCap) (Harris *et al*., 2009). After the study psychiatrist confirms eligibility, the study coordinator, who is independent of administering interventions, will action the randomisation module, allocating the treatment group.

#### Blinding

The statisticians and an independent assessor of symptom severity will be blinded to treatment assignments and will not be permitted access to the randomisation module. Videos for independent assessment of symptom severity will be allocated via a random number generator. If there is an emergency requiring knowledge of a participant’s allocation, the blind may be broken for that individual participant.

### Interventions

#### Intervention description

Following enrolment, all participants will undertake a series of baseline measures, including self-reported outcomes, clinician-rated assessments of motor function and FND symptom severity, treatment expectations, a force-matching task, and an fMRI scan.

Eligible participants will undertake a psilocybin preparation session with the study psychiatrist consistent with existing safety guidelines for psychedelic research (Johnson *et al*., 2008). This will involve building rapport and trust, providing education about psilocybin, intention setting, reviewing relaxation strategies, and advice for preparation, dosing, and integration.

##### Treatment

Participants will then be scheduled for their study treatment. This will comprise two initial physiotherapy sessions, the psilocybin dosing session, and then six physiotherapy sessions within three weeks post-dosing.

##### Physiotherapy

The specialist physiotherapy is based on the Physio4FMD manual used in previous studies in motor FND (Nielsen & Holt, 2024). A workbook will be completed by both the participant and the physiotherapist to help guide the intervention.

The initial two physiotherapy sessions pre-dosing will involve:

- Comprehensive assessment.
- Development of a treatment plan.
- Education on FND following a standardised biopsychosocial model.

The remaining six sessions post-dosing will comprise:

- Video analysis of movement.
- Posture and movement retraining – aiming to redirect attention away from the body and promote positive experiences of symptom-free and automatic, normal movement.
- Advice for managing common co-existing difficulties in FND, such as pain, fatigue, and memory difficulties.
- Codeveloping a self-management plan.

##### Psilocybin dosing session

The psilocybin dosing session will take place within three days of the initial physiotherapy sessions. Upon arrival, the study psychiatrist will ensure the participant’s continued eligibility and safety by reviewing their medications, measuring vital signs, and conducting a urine drug screen and urine pregnancy test (if applicable). The psychiatrist will then administer the prescribed psilocybin. The participant will be encouraged to relax, with the study psychiatrist available to provide support. Vital signs will be rechecked at 0.5-, 1, 3, and 5 hours post-dose, and AEs monitored throughout. For participants randomised to the moderate-dose arm, the physiotherapist will attend at 1.5 and 4.5 hours post-dose to administer a series of movement tasks and any further physiotherapy as tolerated by the participant to explore any potential treatment benefit of novel experiences of movement during the acute drug effects.

The participant will remain under the supervision of the study psychiatrist or physiotherapist for at least 5 hours post-dose and until the acute drug effects have subsided. The participant will then complete questionnaires regarding the intensity of their experience before being taken home by a support person or taxi. The psychiatrist will telephone the participant the following day to invite any further reflections and monitor safety.

#### Follow-up

After the final physiotherapy treatment session, the participant will complete the scheduled outcome measures. Follow-up physiotherapy and psychiatry visits will be scheduled for one week and four weeks post-treatment. Follow-up physiotherapy visits will review any areas of difficulty since completing the treatment, the self-management plan, and the goals. Follow-up psychiatry visits will cover psychological integration post-dosing, AEs, mental health, FND symptoms, and any corresponding changes in functioning. The psychiatrist will also conduct a semi-structured, audio-recorded qualitative interview at the one-week follow-up visit. All scheduled outcome measures will be completed at each of these study visits.

Following study exit, participants will return to the care of their usual treating practitioners, and any relevant handovers will be provided by the study psychiatrist and physiotherapist.

#### Discontinuing or modifying allocated interventions

Participants may withdraw from the study at any point. Investigators can withdraw participants if it is deemed in their best interest, they engage in protocol deviations placing them at risk, exclusion criteria develop, or an AE occurs that affects their safety.

The Principal Investigator has the right to terminate the study at any time and will make the final decision to terminate the study upon completion. If terminated prematurely, investigators will inform participants promptly, and all requirements regarding the storage and secure destruction of study documents and investigational products will be observed.

#### Concomitant care

##### Contraindicated medications

To be enrolled in the study, participants must not be taking:

- Opioids within 12 hours of psilocybin dosing.
- Antidepressants within five half-lives of their cessation before psilocybin dosing.
- Potent enzyme inducers or inhibitors.
- Drugs with a narrow therapeutic index within 12 hours of psilocybin dosing.
- Nicotine and caffeine within 2 hours before and 6 hours following psilocybin dosing.

##### Concomitant care

Medications will be reviewed at screening and before the dosing session. If a contraindicated medication is identified at the screening visit, a discussion between the investigators, participant, and their treating practitioner will take place to determine if it is appropriate to cease the medication before the dosing session. This will include a plan for reviewing and, if required, restarting the medication post-psilocybin dosing.

For contraindicated antidepressant medications, the recommendation will be to wean gradually to mitigate discontinuation effects, cease at least five half-lives before the scheduled dosing session to avoid interactions, and restart (if required) after completing the physiotherapy treatment to avoid initial medication titration adverse effects during the treatment course.

Participants will be required to suspend external physiotherapy for their motor FND throughout study enrolment until their study exit.

#### Outcomes

**Figure 2.**
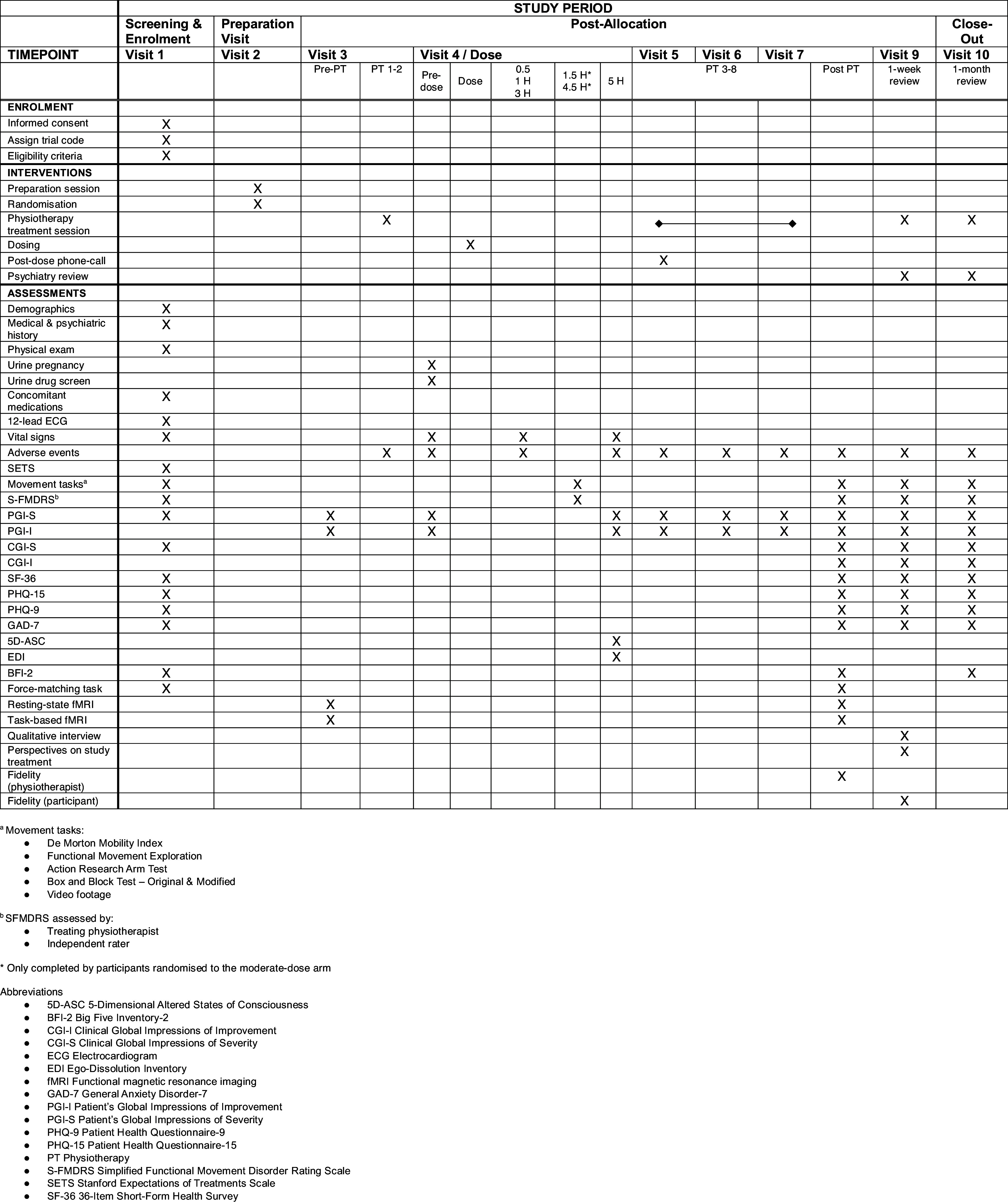
Study schedule.

Time points for all outcome measures are outlined in Fig. 2, and their details are provided in Table 2.

**Table 1.** Eligibility Criteria

Inclusion criteria	Exclusion criteria
Adults aged 18 to 65 years. A diagnosis of refractory motor FND, supported by relevant neurological investigations and independent assessment by a psychiatrist and neurologist. Motor FND is defined as upper or lower limb motor weakness, gait disorder, or movement disorder (e.g., tremor or dystonia) of at least six months duration. Symptoms are deemed refractory if these are of at least six months duration, persist following physiotherapy and psychiatry management, and are considered by the participant to be impacting their functioning or quality of life. Understanding of their diagnosis of FND. Capacity to provide informed consent for the study.	Medical Exclusion Criteria Cardiovascular conditions: poorly-controlled hypertension, angina, ischaemic heart disease, a clinically significant ECG abnormality, transient ischaemic attack, stroke, or peripheral or pulmonary vascular disease. A diagnosis of epilepsy or previous epileptic seizures. A diagnosis of dementia. Chronic kidney or liver disease. Known conditions putting the participant at risk for hypercalcaemia, Cushing’s syndrome, hypoglycaemia, syndrome of inappropriate antidiuretic hormone secretion, or carcinoid syndrome. Insulin-dependent diabetes; if taking oral hypoglycaemic agents, the participant is only excluded if they also have a history of hypoglycaemia. Females who are pregnant, nursing, or trying to conceive. Use of medications contraindicated with psilocybin, that are inappropriate to cease for the necessary period surrounding the dosing sessions. See section ‘Contraindicated Medications’ for details. Enrolled in another clinical trial involving an investigational product. Psychological Exclusion Criteria Current or previous psychotic disorder, including schizophrenia, schizoaffective disorder, schizophreniform disorder, brief psychotic disorder, delusional disorder, schizotypal personality disorder, substance/medication-induced psychotic disorder, or psychotic disorder due to another medical condition. Current or previous bipolar I or II disorder. First-degree relative with a psychotic or bipolar disorder. A history of attempted suicide or mania. Current or previous substance use disorder (excluding caffeine and nicotine). Previous regular use, or current use, of psychedelic agents. Other psychiatric conditions deemed by research staff to be incompatible with safe exposure to psilocybin.

Abbreviations: ECG, Electrocardiogram; FND, Functional neurological disorder.

**Table 2.** Primary, Secondary, and Exploratory Outcomes

	Domain	Measures	Administration	Application and clinical relevance
**Primary Outcomes**	Tolerability	Vital signs	Clinician-administered	Heart rate, blood pressure, and oxygen saturation on room air will be monitored.
		Adverse events form	Clinician-administered	Treatment-emergent adverse events will be recorded in the adverse events form. These will be classified based on the type, number, severity, relatedness to the study drug, and whether the event constitutes a serious adverse event or significant safety issue.
	Feasibility	Completion of movement tasks	Clinician-administered	The S-FMDRS (Nielsen *et al.,* 2017a), DEMMI (de Morton *et al*., 2008), FME, ARAT (Yozbatiran *et al*., 2008), and BBT (Mathiowetz *et al*., 1985) (original and modified versions) measure several domains of motor function to assess the feasibility of competing movement tasks during the acute drug effects.
	Symptom severity	S-FMDRS	Clinician-administered	9-item instrument assessing seven body regions and two functional movements commonly affected in motor FND (Nielsen *et al.,* 2017a). Severity and duration are each rated from 0 to 3 for each of the nine items, with scores summed to provide a total score between 0 to 54 (higher scores indicate greater impairment).
		PGI-S & -I	Self-administered	PGI-S: 1-item instrument, scored from 1 to 4 (higher scores indicate greater severity), assessing disease severity (Food & Drug Administration, 2018). PGI-I: 1-item instrument rated from 1 to 7 (lower scores indicate greater improvement and higher scores indicate worsening) assessing change following an intervention (Mohebbi *et al*., 2018; Remigio-Baker *et al*., 2024).
**Secondary Outcomes**	Symptom severity	S-FMDRS – video assessment	Clinician-administered	Clinician-rated severity of functional motor symptoms, graded through video assessment by a blinded, independent assessor.
		CGI-S & -I	Clinician-administered	CGI-S: 1-item instrument, scored from 1 to 7, assessing disease severity (higher scores indicate greater severity) (Busner & Targum, 2007). CGI-I: 1-item instrument rated from 1 to 7 assessing change following an intervention (lower scores indicate greater improvement and higher scores indicate worsening) (Busner & Targum, 2007).
	Co-occurring symptoms	PHQ-9	Self-administered	9-item scale, scored from 0 to 3, assessing the severity of depressive symptoms over the past two weeks (higher scores indicating greater frequency of symptoms) (Kroenke *et al*., 2001). Items are summed to provide a total score between 0 to 27. An additional 10^th^ item is provided to ascertain the impact on functioning, scored 1 to 4 (higher scores indicating greater impairment).
		GAD-7	Self-administered	7-item scale, scored from 0 to 3, assessing the severity of anxiety symptoms over the past two weeks (higher scores indicating greater frequency of symptoms) (Spitzer *et al*., 2006). Items are summed to provide a total score between 0 to 21. An additional 8^th^ item is provided to ascertain the impact on functioning, scored 1 to 4 (higher scores indicating greater impairment).
		PHQ-15	Self-administered	15-item scale, scored from 0 to 2, assessing the impact of various somatic symptoms over the past week (higher scores indicate greater impact) (Kroenke *et al*., 2002). Items are summed to provide a total score between 0 to 30.
	Quality of life	SF-36	Self-administered	36-item scale, grouped into 8 subscales representing core health-related quality of life dimensions (physical functioning, role limitations due to physical health, pain, general health, emotional wellbeing, social functioning, role limitations due to emotional problems, energy/fatigue, and mental health) (Ware *et al*., 1993). Item responses range from the lowest option at 1 to the highest option from 2 to 6 (depending on the item), with reverse scoring for some items. Each item is then converted to a 0 to 100 range, with higher scores indicating a more favourable health state.
	Treatment expectations	SETS	Self-administered	6-item scale, 3 each for positive and negative expectations, and each item scored on a 7-point Likert scale (lower scores indicate greater disagreement and higher scores indicate greater agreement) (Younger *et al*., 2012).
	Drug intensity	5D-ASC	Self-administered	94-item scale across 11 subscales (de Deus Pontual *et al*., 2023). Responses are rated on a visual analog scale, from ‘No, not more than usually’ to ‘Yes, much more than usually’. Items are summed for each subscale to provide a total score. Higher scores indicate greater alterations in states of consciousness.
		EDI	Self-administered	8-item focused assessment of the experience of ego-dissolution (Nour *et al*., 2016). Each item is rated on a visual analog scale from ‘No, not more than usually’, to ‘Yes, entirely or completely’. Scores are provided for each item and summed to provide a total score. Higher scores indicate greater ego-dissolution.
**Exploratory Outcomes**	Movement tasks	DEMMI	Clinician-administered	15-item unidimensional instrument that assesses mobility from bed-bound to independent mobility, including balance, gait, seating, and strength (de Morton *et al*., 2008). Eleven items are dichotomous (scored 0 or 1), and four items have three response options (scored 0, 1, or 2) based on ability and time to completion (lower scores indicate greater impairment). Raw scores (from 0 to 19) are converted to a total DEMMI score (0 to 100) as defined by the scoring key. Additional comments for duration are provided where relevant.
		FME	Clinician-administered	FND extension module developed by our team, assessing movements incorporated in the physiotherapy intervention used in this study (Nielsen & Holt, 2024), to assess additional movements, including standing up from a chair, weight shifting, and balance. Items are dichotomous (scored 0 or 1) based on ability (lower scores indicate greater impairment). Items are summed to provide a total score between 0 to 4. Additional comments for duration are provided where relevant.
		ARAT	Clinician-administered	19-item instrument assessing upper limb coordination, dexterity, and functioning (lower scores indicate greater impairment) (Yozbatiran *et al*., 2008). Items are scored from 0 to 3 (lower scores indicate greater impairment). Scores are provided for four subscales, defined as grasp (sum of six items, range 0 to 18), grip (sum of four items, range 0 to 12), pinch (sum of six items, range 0 to 18), and gross movement (sum of three items, range 0 to 9). Items are summed to provide a total score from 0 to 57.
		BBT – original and modified	Clinician-administered	Original – movement of blocks from one box to another over 60 s, assessing unilateral gross manual dexterity. It has shown high test–retest validity from normative data in adults without disability (Mathiowetz *et al*., 1985). Modified – as above, but with a shield placed between the participant’s vision and their hands and access to a mirror to view their movements via reflection. This modulates attentional mechanisms of movement – an important feature of motor FND management. Lower scores for each scale are indicative of greater impairment. Lower scores for each scale are indicative of greater impairment.
		Video footage	Clinician-administered	Review of video footage of movement tasks to assess movement quality.
	Sensorimotor function	Force-matching task	Clinician-administered	Force-matching performance and sensory attenuation caused by self-generated movement (Pareés *et al*., 2014).
	Brain activity	Resting-state fMRI	Clinician-administered	Resting-state measures of brain activity (McCulloch *et al*., 2022).
		Task-based fMRI	Clinician-administered	Measures of brain activity during a Bayesian-belief updating task (Sharot *et al*., 2011; Korn *et al*., 2014; Marks & Baines, 2017).
	Personality	BFI-2	Self-administered	60-item scale based on the five-factor model of personality (Soto & John, 2017). Items are scored on a 5-point Likert scale (lower scores indicate greater disagreement and higher scores indicate greater agreement, with reverse option wording across some items). Items are summed for 5 domains and 15 facet subscales.
	Subjective experiences	Perspectives on study treatment	Self-administered	9-item questionnaire including responses on ordinal scales, a visual analog scale, and free-text, exploring perspectives on aspects of the study treatment, including physiotherapy and psilocybin.
		Qualitative interview	Self-administered	Qualitative interview exploring subjective experiences of psilocybin, completing movement tasks during the acute drug effects, and the study treatment.

Abbreviations: 5D-ASC, 5-Dimensional Altered States of Consciousness; ARAT, Action Research Arm Test; BBT, Box and Block Test; BFI-2, Big Five Inventory-2; CGI-I, Clinical Global Impressions of Improvement; CGI-S, Clinical Global Impressions of Severity; DEMMI, De Morton Mobility Index; EDI, Ego-Dissolution Inventory; FME, Functional Movement Exploration; fMRI, Functional magnetic resonance imaging; GAD-7, General Anxiety Disorder-7; PGI-I, Patient’s Global Impressions of Improvement; PGI-S, Patient’s Global Impressions of Severity; PHQ-9, Patient Health Questionnaire-9; PHQ-15, Patient Health Questionnaire-15; S-FMDRS, Simplified Functional Movement Disorder Rating Scale; SETS, Stanford Expectations of Treatments Scale; SF-36, 36-Item Short-Form Health Survey.

##### Primary outcomes

Tolerability will be assessed by checking vital signs regularly during the dosing sessions and recording AEs arising at any time during the study. AEs will be classified based on the type, number, severity, relatedness to the study drug, and whether the event constitutes a serious adverse event (SAE) or significant safety issue (SSI).

The feasibility of participating in physiotherapy during the acute effects of psilocybin 15 mg will be assessed by measuring the successful completion of a series of movement tasks. These will include the S-FMDRS (Nielsen *et al.,* 2017a), DEMMI (de Morton *et al*., 2008), FME, ARAT (Yozbatiran *et al*., 2008), and BBT (Mathiowetz *et al*., 1985) (original and modified versions) at 1.5 and 4.5 hours post-dosing.

The S-FMDRS assesses seven body regions and two functional movements (gait and speech) commonly affected by motor FND. Severity and duration are each rated from 0 to 3 for each of the nine items (higher scores indicate greater impairment), with items summed to provide a total score between 0 and 54. The scale may be completed by neurologists and physiotherapists, has high inter-rater reliability for the total score, and is sensitive to treatment-related changes. The assessment of the S-FMDRS by the study physiotherapist will comprise the primary outcome for assessing clinician-rated symptom severity. The S-FMDRS will be completed at baseline, 1.5 and 4.5 hours post-dosing for participants receiving psilocybin 15 mg, completion of the physiotherapy treatment, and the one-week and four-week follow-up visits.

The PGI-S and PGI-I are self-rated, single-item scales to capture static and dynamic aspects of disease severity and change, respectively (Food and Drug Administration, 2018). The PGI-S is rated from 1 to 4 (higher scores indicate greater impairment), and the PGI-I is rated from 1 to 7 (lower scores indicate greater improvement, and higher scores indicate worsening). These measures have shown satisfactory feasibility, validity, and sensitivity to treatment-related changes in studies on neuropsychiatric symptoms (Mohebbi *et al*., 2018; Snyder *et al*., 2021; Remigio-Baker *et al*., 2024). The PGI-S will be completed at baseline, and both scales completed on the morning of each day of the physiotherapy treatment and dosing session, following completion of the physiotherapy treatment, and at the one-week and four-week follow-up visits.

##### Secondary outcomes

An independent assessment of each S-FMDRS measure, as captured using video footage, will be completed by the study neurologist who will be blinded to treatment allocation. This will comprise a secondary outcome to help assess the interrater reliability and utility of this measure for this and subsequent studies. To complement the participant-reported PGI-I and PGI-S scales, the clinician-rated CGI-I and CGI-S (Busner & Targum, 2007) will be administered to objectively assess disease severity and change, respectively, post-treatment.

Depressive symptoms will be measured by the PHQ-9 (Kroenke *et al*., 2001), anxiety symptoms by the GAD-7 (Spitzer *et al*., 2006), somatic symptoms by the PHQ-15 (Kroenke *et al*., 2002), and health-related quality of life by the SF-36 (Ware *et al*., 1993). Treatment expectations will be assessed pre-dosing by the SETS (Younger *et al*., 2012), and intensity of the acute drug effects assessed post-dosing by the 5D-ASC (de Deus Pontual *et al*., 2023) and EDI (Nour *et al*., 2016).

##### Exploratory outcomes

Video footage and individual scores for each of the DEMMI, FME, ARAT, and BBT (original and modified versions) will be assessed to explore the impact of psilocybin-assisted physiotherapy on several domains of motor function. Resting-state fMRI will be used to investigate alterations in large-scale brain networks and potential treatment mechanisms (Daws *et al*., 2022; Berkovitch *et al*., 2025). This will be complemented by a Bayesian belief updating fMRI task, which will assess changes in optimism bias and any underlying neuroimaging correlates (Sharot *et al*., 2011; Korn *et al*., 2014; Marks & Baines, 2017). This task has revealed differences between healthy subjects and patients with MDD and is of relevance given the hypothesised role of aberrant predictive processing in motor FND. Given previous findings of reduced sensory attenuation in motor FND, which may arise through related mechanisms, treatment-induced normalisation of sensorimotor performance using a force-matching task will also be assessed (Pareés *et al*., 2014). In line with previous studies demonstrating changes in personality following psychedelics, such as increased ‘openness’, and the relevance of these changes to therapeutic effects, personality traits will be measured via the BFI-2 (Soto & John, 2017). Subjective experiences will also be assessed by surveying participant perspectives on the intervention and conducting a qualitative interview following the treatment.

## Data collection, management, and analysis

### Data collection and assessment

The study physiotherapist will provide the physiotherapy treatment and physiotherapy follow-up visits, administer movement tasks, and assess symptom severity. The study neurologist will conduct independent assessments of the S-FMDRS, as captured using video footage. Qualified radiographers will conduct fMRI scans. The study psychiatrists will conduct the screening visit, preparation session, dosing session, post-dosing phone call, psychiatry follow-up visits, qualitative interview, and remaining scheduled measures at these visits. At all times, the study team member administering the assessment has full responsibility for the accuracy, completeness, and timeliness of all data captured.

### Data management

The majority of study data will be collected and managed using REDCap (Harris *et al*., 2009) – a secure, browser-based application for managing online surveys and databases – hosted at the University of Melbourne. Scanned paper-based Case Report Forms (CRFs), video footage, recordings and transcripts of qualitative interviews, and fMRI images will be uploaded and stored in secure, password-protected servers hosted by the University of Melbourne and available only to permitted trial staff. Results from urine tests will be recorded, and urine samples will be disposed of in biological hazard waste bins.

Confidentiality will be maintained by assigning participants a unique code to record any data collected, and paper-based CRFs will be stored in locked filing cabinets. Videos of physiotherapy task performance will be de-identified using facial blurring, and qualitative interview audio recordings will be transcribed in a de-identified manner.

On study completion, scanned and electronic source documents will be archived on password-protected servers, and paper-based CRFs kept in locked cabinets. All CRFs will be retained for 15 years and then destroyed by secure shredding and deletion from protected servers according to the University of Melbourne records management policy.

Data management will be carried out to a standard of security and confidentiality consistent with Good Clinical Practice (International Council for Harmonisation, 2015). Data will be handled only by the research team and held at the Department of Psychiatry, University of Melbourne, Austin Health.

### Adherence and retention

Before their study treatment, participants will receive a detailed appointment handout, which also reiterates pre- and post-dosing requirements and recommendations. Travel costs incurred for attending each study visit will be reimbursed, up to $50 per visit, and food and drink will be provided at the dosing session. No additional financial inducements will be provided. The psychiatry follow-up visits and qualitative interview will be offered either in person or via video call, as per participant preference, to promote retention by reducing the required number of in-person visits. The study team will monitor data in real time to ensure completeness and will document attempts to obtain follow-up data and any protocol deviations.

To assess the fidelity of the physiotherapy treatment (the extent to which the treatment followed the intervention protocol):

1. The study physiotherapist will complete a checklist for each participant, based on the template for intervention description and replication (TIDieR) checklist description (Hoffmann *et al*., 2014).
2. The content, length, and number of physiotherapy sessions by participant report will be monitored with a structured telephone survey following completion of the physiotherapy treatment.
3. A random sample of completed physiotherapy workbooks will be assessed by an independent physiotherapist against predetermined criteria.

### Statistical methods

Prior to the study database being locked, a comprehensive statistical analysis plan will be finalised. The analysis will encompass all participants who were randomised, regardless of whether they completed their full study enrolment. All available data from these participants will be included. The reasons for any participant’s early withdrawal from the study will be documented.

An interim analysis using unblinded data will be performed after 50% (*n* = 12) of the target sample size has completed their study enrolment. This analysis will consist of summary statistics of tolerability and efficacy outcomes by treatment groups. These interim results will support planning for a follow-up RCT in motor FND participants. As such, the goal of the interim analysis is not related to this pilot study, and no sample size adjustment will be made as a result. Blinding will be maintained as described earlier.

## Monitoring

The trial management group (TMG) will comprise all authors listed and provide overall supervision of the trial, including protocol development, oversight of trial progress, and publication and dissemination of trial results. A data monitoring committee is not needed for this study because of the limited known risks, short study duration, and Phase 1 objectives centred on feasibility and safety.

## Adverse event reporting and harms

Common acute AEs reported following psilocybin administration included headache, nausea, anxiety, dizziness, and blood pressure elevations and were mostly mild-to-moderate in severity and transient (Johnson *et al*., 2012; Breeksema *et al*., 2022; Yerubandi *et al*., 2024). When incorporating long-term outcomes, contemporary clinical studies revealed no reports of death by suicide, persisting psychotic disorder, or hallucinogen persistent perceptual disorder (Hinkle *et al*., 2024). However, concerns for incomplete reporting and identification have been raised, and further research is required into the preferred management of AEs and long-term safety (Hinkle *et al*., 2024; Simonsson *et al*., 2024). Therefore, safeguards will be implemented throughout this study to comprehensively monitor, report, and respond to safety concerns.

During psilocybin dosing, the study psychiatrist will be available to respond to any psychological distress. If severe, benzodiazepines and antipsychotics will be available for administration. A Medical Emergency Team notification will be initiated if these measures are ineffective, heart rate exceeds 130 beats per minute, systolic blood pressure exceeds 180 mmHg, oxygen saturation falls below 90%, there is a reduced level of consciousness, or symptoms arise that warrant urgent medical assessment. The site physicians will be available via mobile phone throughout enrolment.

All AEs will be monitored by the study physicians until resolution or, if unresolved or chronic, further follow-up is arranged as warranted. All SAEs and SSIs will be reported as per Austin Health’s safety reporting policy.

## Dissemination plans

Results will be submitted to peer-reviewed journals for publication and presented at psychiatry, neurology, physiotherapy, or other relevant conferences.

De-identified safety data will be shared with the Usona Institute. Access to the full trial dataset will only be available to investigators and any other relevant regulatory bodies. Study intellectual property arising from this study will be owned by the University of Melbourne.

## Discussion

Conceptual frameworks for FND have evolved from exclusively psychological to biopsychosocial models, supported by evidence implicating physiological and sensorimotor processes (Drane *et al*., 2021; Pareés *et al*., 2014; Ricciardi *et al*., 2016; Perez *et al*., 2021; Raynor & Baslet, 2021; Sojka *et al*., 2021). This shift in understanding is reflected in expert consensus recommendations of physiotherapy as part of multidisciplinary treatment of motor FND (Nielsen *et al*., 2015). However, the limited efficacy of existing interventions and the impact of the disorder emphasise the need for new treatments that target other aspects of FND pathophysiology. Research into psychedelics has demonstrated encouraging potential in several neuropsychiatric disorders, and this pilot study was conceptualised based on these advances in the pathophysiological understanding of motor FND and hypothesised treatment mechanisms of psychedelics.

Given the uncertainties regarding the therapeutic mechanisms of psychedelics and the most appropriate treatment model, this study will therefore compare additional active physiotherapy during the acute effects of moderate-dose psilocybin versus a more conventional, standard-dose PAT approach.

This study will enrol participants with treatment-refractory motor FND. This provides an opportunity to evaluate the potential added benefit of psychedelic treatment in those who have already received the current gold-standard physiotherapy approach. The comprehensive screening and eligibility criteria in this trial will help exclude participants at greater risk of harm following psychedelic treatment (MacCallum *et al*., 2022). The regular safety reporting, psychiatric support, and availability of study physicians throughout the study will enable prompt action to address any concerns arising and provide a greater understanding of the tolerability of this intervention.

The wide range of validated outcome measures spanning motor function, psychiatric and physical symptoms, and quality of life domains will enable assessment of benefits beyond core symptom relief and the potential for this treatment to address shared biopsychosocial factors across these areas (Butler *et al*., 2021). Exploratory outcomes examining underlying mechanisms, such as fMRI and the force-matching task, may deepen these insights and identify potential biomarkers and mediators of treatment response. The inclusion of both clinician-rated and self-rated outcomes, including qualitative interviews, will facilitate balanced inquiries into objective and subjective changes. The utility of these measures will also be assessed for their consideration in future, adequately powered studies.

These findings may inform psychedelic-assisted therapy studies in other FND subtypes, such as functional seizures, and related neuropsychiatric disorders with shared pathophysiological mechanisms, such as chronic pain, fibromyalgia, and chronic fatigue syndrome (Castellanos *et al*., 2020; Glynos *et al*., 2023; Wilde, 2023; Butler *et al*., 2024). Beyond the rationale for combining psychedelics with physiotherapy for motor FND, there exists a theoretical basis for the use of psychoplastogens in treating other neuropsychiatric disorders associated with motor dysfunction, such as stroke and acquired brain injury (Nardou *et al*., 2023; Allen *et al*., 2024; Yang *et al*., 2025). This is of considerable interest given the known impact of psychedelics upon perceptual function closely related to motor control, and this will be the first study combining psychedelics with movement retraining in a clinical population, providing valuable insights into the feasibility of this approach in these related conditions.

While not investigated in this pilot study, a future consideration is the role of psychedelic microdosing. This involves consumption of ‘sub-perceptual’ psychedelic doses with purported cognitive and therapeutic benefits (Andersson & Kjellgren, 2019; Fadiman & Korb, 2019; Lea *et al*., 2020). However, evidence for micro-dosing is constrained by a lack of controlled studies (Polito & Liknaitzky, 2022; Murphy *et al*., 2024) and the reduced psychoplastogenic effects at these low doses (Jefsen *et al*., 2021; Barksdale *et al*., 2024). Nevertheless, it is possible that this treatment paradigm may be worth exploring in the future as the field evolves.

Given this study’s primary objectives focus on tolerability and feasibility, the therapeutic outcomes will be limited by the open-label design, small sample size, and relatively short follow-up. We also acknowledge challenges arising via the temporary cessation of psychotropic medications, including ongoing uncertainty and study into how best to incorporate these pre-existing medications with psychedelic treatment (Goodwin *et al*., 2023; Tap *et al*., 2025).

This protocol outlines the first study of psychedelic treatment for motor FND in over 50 years. The study design builds upon recent findings of the safety and feasibility of performing physiotherapy following psilocybin administration in healthy participants. The results from this study will inform the design of a planned, adequately powered RCT of psilocybin-assisted physiotherapy in motor FND.

## Trial registration

Trial registered on Australian New Zealand Clinical Trials Registry (ANZCTR).

Registration Number: ACTRN12621000578808

Date Registered: 17 May 2021

URL: https://www.anzctr.org.au/Trial/Registration/TrialReview.aspx?id=381745

## Protocol version

1 April 2025

Version 6.0

Identifier: 2025_APR_01 PROTOCOL_PsyFND_V6

## References

[ref1] Allen J , Dames SS , Foldi CJ and Shultz SR (2024) Psychedelics for acquired brain injury: a review of molecular mechanisms and therapeutic potential. Molecular Psychiatry 29(3), 671–685. 38177350 10.1038/s41380-023-02360-0

[ref2] Andersen KAA , Carhart-Harris R , Nutt DJ and Erritzoe D (2021) Therapeutic effects of classic serotonergic psychedelics: a systematic review of modern-era clinical studies. Acta Psychiatrica Scandinavica 143(2), 101–118. 33125716 10.1111/acps.13249

[ref3] Andersson M and Kjellgren A (2019) Twenty percent better with 20 micrograms? a qualitative study of psychedelic microdosing self-rapports and discussions on youTube. Harm Reduction Journal 16, 63. 31779667 10.1186/s12954-019-0333-3 PMC6883685

[ref4] Aybek S and Perez DL (2022) Diagnosis and management of functional neurological disorder. The BMJ 376, o64. 35074803 10.1136/bmj.o64

[ref5] Barber GS and Aaronson ST (2022) The emerging field of psychedelic psychotherapy. Current Psychiatry Reports 24(10), 583–590. 36129571 10.1007/s11920-022-01363-y PMC9553847

[ref6] Barksdale BR , Doss MK , Fonzo GA and Nemeroff CB (2024) The mechanistic divide in psychedelic neuroscience: an unbridgeable gap? Neurotherapeutics 21(2), e00322. 38278658 10.1016/j.neurot.2024.e00322 PMC10963929

[ref7] Berkovitch L , Fauvel B , Preller KH and Gaillard R (2025) Neurocognitive effects of psilocybin: a systematic and comprehensive review of neuroimaging studies in humans. Neuroscience and Biobehavioral Reviews 175, 106239. 40456393 10.1016/j.neubiorev.2025.106239

[ref8] Bhagavan C , Kanaan R , Carter O , Nielsen G , Berlowitz D , Issak S , Braat S , Zaloumis S , Attard Z , Oliver G , Mayne D , McKernon D , Roebuck G , Rucker J , Butler M and Bryson A (2024) Psilocybin and motor function: a triple-blind, dose-finding study in healthy participants. Psychiatric Research and Clinical Practice 6, 164–174. 39669539 10.1176/appi.prcp.20240047 PMC11633464

[ref9] Binzer M , Andersen PM and Kullgren G (1997) Clinical characteristics of patients with motor disability due to conversion disorder: a prospective control group study. Journal of Neurology, Neurosurgery & Psychiatry 63, 83–88. 9221972 10.1136/jnnp.63.1.83 PMC2169635

[ref10] Breeksema JJ , Kuin BW , Kamphuis J , van den Brink W , Vermetten E and Schoevers RA (2022) Adverse events in clinical treatments with serotonergic psychedelics and MDMA: a mixed-methods systematic review. Journal of Psychopharmacology 36(10), 1100–1117. 36017784 10.1177/02698811221116926 PMC9548934

[ref11] Bryson A , Carter O , Norman T and Kanaan R (2017) 5-HT2A agonists: a novel therapy for functional neurological disorders? International Journal of Neuropsychopharmacology 20, 422–427. 28177082 10.1093/ijnp/pyx011 PMC5417053

[ref12] Busner J and Targum SD (2007) The clinical global impressions scale: applying a research tool in clinical practice. Psychiatry (Edgmont) 4, 28. PMC2880930 20526405

[ref15] Butler M , Bird C , Maggio C , Durden A , Modlin N , Campbell-Coker K , Edwards M , Pick S , Millman L.S. M , Lowery E , Bhagavan C , Kanaan R , Golder D , Mildon B , Mehta M , Rucker J and Nicholson TR (2024) Probing the functional magnetic resonance imaging response to psilocybin in functional neurological disorder (PsiFUND): study protocol. Wellcome Open Research 9, 401. 39372842 10.12688/wellcomeopenres.22543.2 PMC11450546

[ref13] Butler M , Seynaeve M , Nicholson TR , Pick S , Kanaan RA , Lees A , Young AH and Rucker J (2020) Psychedelic treatment of functional neurological disorder: a systematic review. Therapeutic Advances in Psychopharmacology 10, 2045125320912125. 32435447 10.1177/2045125320912125 PMC7225815

[ref14] Butler M , Shipston-Sharman O , Seynaeve M , Bao J , Pick S , Bradley‐Westguard A , Ilola E , Mildon B , Golder D , Rucker J and Stone J (2021) International online survey of 1048 individuals with functional neurological disorder. European Journal of Neurology 28, 3591–3602. 34245646 10.1111/ene.15018

[ref16] Carhart-Harris RL and Friston KJ (2019) REBUS and the anarchic brain: toward a unified model of the brain action of psychedelics. Pharmacological Reviews, American Society for Pharmacology and Experimental 71, 316–344. 10.1124/pr.118.017160 PMC6588209 31221820

[ref17] Castellanos JP , Woolley C , Bruno KA , Zeidan F , Halberstadt A and Furnish T (2020) Chronic pain and psychedelics: a review and proposed mechanism of action. Regional Anesthesia and Pain Medicine 45(7), 486–494. 32371500 10.1136/rapm-2020-101273

[ref18] Crimlisk HL , Bhatia KP , Cope H , David AS , Marsden D and Ron MA (2000) Patterns of referral in patients with medically unexplained motor symptoms. Journal of Psychosomatic Research 49, 217–219. 11110993 10.1016/s0022-3999(00)00167-7

[ref19] Czarnecki K , Thompson JM , Seime R , Geda YE , Duffy JR and Ahlskog JE (2012) Functional movement disorders: successful treatment with a physical therapy rehabilitation protocol. Parkinsonism & Related Disorders 18, 247–251. 22113131 10.1016/j.parkreldis.2011.10.011

[ref20] Dallocchio C , Tinazzi M , Bombieri F , Arnó N and Erro R (2016) Cognitive behavioural therapy and adjunctive physical activity for functional movement disorders (Conversion disorder): a pilot, single-blinded, randomized study. Psychotherapy and Psychosomatics 85(6), 381–383. 27744440 10.1159/000446660

[ref21] Daws RE , Timmermann C , Giribaldi B , Sexton JD , Wall MB , Erritzoe D , Roseman L , Nutt D and Carhart-Harris R (2022) Increased global integration in the brain after psilocybin therapy for depression. Nature Medicine 28, 844–851. 10.1038/s41591-022-01744-z 35411074

[ref22] de Deus Pontual AA , Senhorini HG , Corradi-Webster CM , Tófoli LF and Daldegan-Bueno D (2023) Systematic review of psychometric instruments used in research with psychedelics. Journal of Psychoactive Drugs 55(3), 359–368. 35616606 10.1080/02791072.2022.2079108

[ref23] de Morton NA , Davidson M and Keating JL (2008) The de morton mobility index (DEMMI): an essential health index for an ageing world. Health and Quality of Life Outcomes 6, 63. 18713451 10.1186/1477-7525-6-63 PMC2551589

[ref24] Drane DL , Fani N , Hallett M , Khalsa SS , Perez DL and Roberts NA (2021) A framework for understanding the pathophysiology of functional neurological disorder. CNS Spectrums 26, 555–561. 10.1017/S1092852920001789 PMC7930164 32883381

[ref25] Edwards MJ , Adams RA , Brown H , Pareés I and Friston KJ (2012) A Bayesian account of ‘hysteria’. Brain 135, 3495–3512. 22641838 10.1093/brain/aws129 PMC3501967

[ref26] Fadiman J and Korb S (2019) Might microdosing psychedelics be safe and beneficial? an initial exploration. Journal of Psychoactive Drugs 51, 118–122. 30925850 10.1080/02791072.2019.1593561

[ref27] Food and Drug Administration (2018) Patient-focused drug development guidance public workshop: methods to identify what is important to patients & select, develop or modify fit-for-purpose clinical outcomes assessments. Maryland: Food and Drug Administration.

[ref28] Friston K (2010) The free-energy principle: a unified brain theory? Nature Reviews Neuroscience 11(2), 127–138. 20068583 10.1038/nrn2787

[ref29] Gelauff J , Stone J , Edwards M and Carson A (2014) The prognosis of functional (psychogenic) motor symptoms: a systematic review. Journal of Neurology, Neurosurgery and Psychiatry 85, 220–226. 24029543 10.1136/jnnp-2013-305321

[ref30] Glynos NG , Pierce J , Davis AK , McAfee J and Boehnke KF (2023) Knowledge, perceptions, and use of psychedelics among individuals with fibromyalgia. Journal of Psychoactive Drugs 55, 73–84. 35001856 10.1080/02791072.2021.2022817

[ref31] Goodwin GM , Croal M , Feifel D , Kelly JR , Marwood L , Mistry S , O’Keane V , Peck SK , Simmons H , Sisa C and Stansfield SC (2023) Psilocybin for treatment resistant depression in patients taking a concomitant SSRI medication. Neuropsychopharmacology 48, 1492–1499. 37443386 10.1038/s41386-023-01648-7 PMC10425429

[ref32] Hallett M , Aybek S , Dworetzky BA , McWhirter L , Staab JP and Stone J (2022) .Functional neurological disorder: new subtypes and shared mechanisms. The Lancet Neurology 21(6), 537–550. 35430029 10.1016/S1474-4422(21)00422-1 PMC9107510

[ref33] Harris PA , Taylor R , Thielke R , Payne J , Gonzalez N and Conde JG (2009) Research electronic data capture (REDCap)-a metadata-driven methodology and workflow process for providing translational research informatics support. Journal of Biomedical Informatics 42, 377–381. 18929686 10.1016/j.jbi.2008.08.010 PMC2700030

[ref34] Hinkle JT , Graziosi M , Nayak SM and Yaden DB (2024) Adverse events in studies of classic psychedelics: a systematic review and meta-analysis. JAMA Psychiatry 81, 1225–1235. 39230883 10.1001/jamapsychiatry.2024.2546 PMC11375525

[ref35] Hinson VK , Weinstein S , Bernard B , Leurgans SE and Goetz CG (2006) Single-blind clinical trial of psychotherapy for treatment of psychogenic movement disorders. Parkinsonism and Related Disorders 12, 177–180. 16364676 10.1016/j.parkreldis.2005.10.006

[ref36] Hoffmann TC , Glasziou PP , Boutron I , Milne R , Perera R , Moher D , Altman DG , Barbour V , Macdonald H , Johnston M and Lamb SE (2014) Better reporting of interventions: template for intervention description and replication (TIDieR) checklist and guide. The BMJ 348, g1687. 24609605 10.1136/bmj.g1687

[ref37] Hull M , Parnes M and Jankovic J (2021) Increased incidence of functional (psychogenic) movement disorders in children and adults amid the COVID-19 pandemic: a cross-sectional study. Neurology: Clinical Practice 11, e686–e690. 34840884 10.1212/CPJ.0000000000001082 PMC8610548

[ref38] International Council for Harmonisation. (2015) Integrated addendum to ICH E6 (R1): guideline for good clinical practice E6 (R2). International Council for Harmonisation, Geneva 2, 1–60.

[ref39] Jefsen OH , Elfving B , Wegener G and Müller HK (2021) Transcriptional regulation in the rat prefrontal cortex and hippocampus after a single administration of psilocybin. Journal of Psychopharmacology 35, 483–493. 33143539 10.1177/0269881120959614

[ref41] Johnson MW , Andrew Sewell R and Griffiths RR (2012) Psilocybin dose-dependently causes delayed, transient headaches in healthy volunteers. Drug and Alcohol Dependence 123, 132–140. 22129843 10.1016/j.drugalcdep.2011.10.029 PMC3345296

[ref40] Johnson MW , Richards WA and Griffiths RR (2008) Human hallucinogen research: guidelines for safety. Journal of Psychopharmacology 22, 603–620. 18593734 10.1177/0269881108093587 PMC3056407

[ref42] Jordbru A , Smedstad L , KlungsÃ¸yr O and Martinsen E (2014) Psychogenic gait disorder: a randomized controlled trial of physical rehabilitation with one-year follow-up. Journal of Rehabilitation Medicine 46, 181–187. 24248149 10.2340/16501977-1246

[ref43] Kompoliti K , Wilson B , Stebbins G , Bernard B and Hinson V (2014) Immediate vs. delayed treatment of psychogenic movement disorders with short term psychodynamic psychotherapy: randomized clinical trial. Parkinsonism and Related Disorders 20, 60–63. 24120952 10.1016/j.parkreldis.2013.09.018

[ref44] Korn CW , Sharot T , Walter H , Heekeren HR and Dolan RJ (2014) Depression is related to an absence of optimistically biased belief updating about future life events. Psychological Medicine 44, 579–592. 23672737 10.1017/S0033291713001074 PMC3880066

[ref45] Kroenke K , Spitzer RL and Williams JBW (2001) The PHQ-9: validity of a brief depression severity measure. Journal of General Internal Medicine 16, 606–613. 11556941 10.1046/j.1525-1497.2001.016009606.x PMC1495268

[ref46] Kroenke K , Spitzer RL and Williams JBW (2002) The PHQ-15: validity of a new measure for evaluating the severity of somatic symptoms. Psychosomatic Medicine 64, 258–266. 11914441 10.1097/00006842-200203000-00008

[ref47] Lea T , Amada N and Jungaberle H (2020) Psychedelic microdosing: a subreddit analysis. Journal of Psychoactive Drugs 52, 101–112. 31648596 10.1080/02791072.2019.1683260

[ref48] Lepow L , Morishita H and Yehuda R (2021) Critical period plasticity as a framework for psychedelic-assisted psychotherapy. Frontiers in Neuroscience 15, 710004. 34616272 10.3389/fnins.2021.710004 PMC8488335

[ref49] Ly C , Greb AC , Cameron LP , Wong JM , Barragan EV , Wilson PC , Burbach KF , Soltanzadeh Zarandi S , Sood A , Paddy MR , Duim WC , Dennis MY , McAllister AK , Ori-McKenney KM , Gray JA and Olson DE (2018) Psychedelics promote structural and functional neural plasticity. Cell Reports 23, 3170–3182. 29898390 10.1016/j.celrep.2018.05.022 PMC6082376

[ref50] MacCallum CA , Lo LA , Pistawka CA and Deol JK (2022) Therapeutic use of psilocybin: practical considerations for dosing and administration. Frontiers in Psychiatry 13, 1040217. 36532184 10.3389/fpsyt.2022.1040217 PMC9751063

[ref51] Madsen MK , Stenbæk DS , Arvidsson A , Armand S , Marstrand-Joergensen MR , Johansen SS , Linnet K , Ozenne B , Knudsen GM and Fisher PM (2021) Psilocybin-induced changes in brain network integrity and segregation correlate with plasma psilocin level and psychedelic experience. European Neuropsychopharmacology 50, 121–132. 34246868 10.1016/j.euroneuro.2021.06.001

[ref52] Marks J and Baines S (2017) Optimistic belief updating despite inclusion of positive events. Learning and Motivation 58, 88–101.

[ref53] Mathiowetz V , Volland G , Kashman N and Weber K (1985) Adult norms for the box and block test of manual dexterity. The American Occupational Therapy Association 39, 386–391. 10.5014/ajot.39.6.386 3160243

[ref87] McCulloch DE , Madsen MK , Stenbaek DS , Kristiansen S , Ozenne B , Jensen PS , Knudsen GM and Fisher PM (2022) Lasting effects of a single psilocybin dose on resting-state functional connectivity in healthy individuals. Journal of Psychopharmacology 36(1), 74–84. 34189985 10.1177/02698811211026454 PMC8801642

[ref54] Mohebbi M , Dodd S , Dean O M and Berk M (2018) Patient centric measures for a patient centric era: agreement and convergent between ratings on the patient global impression of improvement (PGI-I) scale and the clinical global impressions – improvement (CGI-S) scale in bipolar and major depressive disorder. European Psychiatry 53, 17–22. 29859377 10.1016/j.eurpsy.2018.05.006

[ref55] Murphy RJ , Muthukumaraswamy S and de Wit H (2024) Microdosing psychedelics: current evidence from controlled studies. Biological Psychiatry: Cognitive Neuroscience and Neuroimaging 9(5), 500–511. 38280630 10.1016/j.bpsc.2024.01.002

[ref56] Nardou R , Sawyer E , Song YJ , Wilkinson M , Padovan-Hernandez Y , De Deus JL , Wright N , Lama C , Faltin S , Goff LA and Stein-O’Brien GL (2023) Psychedelics reopen the social reward learning critical period. Nature 618, 790–798. 37316665 10.1038/s41586-023-06204-3 PMC10284704

[ref60] Nielsen G , Buszewicz M , Stevenson F , Hunter R , Holt K , Dudziec M , Ricciardi L , Marsden J , Joyce E and Edwards MJ (2017b) Randomised feasibility study of physiotherapy for patients with functional motor symptoms. Journal of Neurology, Neurosurgery and Psychiatry 88, 484–490. 27694498 10.1136/jnnp-2016-314408

[ref57] Nielsen G and Holt K (2024) Physio4FMD: a randomised controlled trial of specialist physiotherapy for functional motor disorder. Trial materials. University of London.

[ref59] Nielsen G , Ricciardi L , Meppelink AM , Holt K , Teodoro T and Edwards M (2017a) A simplified version of the psychogenic movement disorders rating scale: the simplified functional movement disorders rating scale (S-FMDRS). Movement Disorders Clinical Practice 4, 710–716. 30363505 10.1002/mdc3.12475 PMC6174502

[ref61] Nielsen G , Stone J , Lee TC , Goldstein LH , Marston L , Hunter RM , Carson A , Holt K , Marsden J , Le Novere M and Nazareth I (2024) Specialist physiotherapy for functional motor disorder in England and Scotland (Physio4FMD): a pragmatic, multicentre, phase 3 randomised controlled trial. The Lancet Neurology 23(7), 675–686. 38768621 10.1016/S1474-4422(24)00135-2

[ref58] Nielsen G , Stone J , Matthews A , Brown M , Sparkes C , Farmer R , Masterton L , Duncan L , Winters A , Daniell L and Lumsden C (2015) Physiotherapy for functional motor disorders: a consensus recommendation. Journal of Neurology, Neurosurgery & Psychiatry 86, 1113–1119. 25433033 10.1136/jnnp-2014-309255 PMC4602268

[ref62] Nour MM , Evans L , Nutt D and Carhart-Harris RL (2016) Ego-dissolution and psychedelics: validation of the ego-dissolution inventory (EDI). Frontiers in Human Neuroscience 10, 190474. 10.3389/fnhum.2016.00269 PMC4906025 27378878

[ref63] Pareés I , Brown H , Nuruki A , Adams RA , Davare M , Bhatia KP , Friston K and Edwards MJ (2014) Loss of sensory attenuation in patients with functional (psychogenic) movement disorders. Brain 137(11), 2916–2921. 25161293 10.1093/brain/awu237

[ref64] Passie T , Guss J and Krähenmann R (2022) Lower-dose psycholytic therapy – a neglected approach. Frontiers in Psychiatry 13, 1020505. 36532196 10.3389/fpsyt.2022.1020505 PMC9755513

[ref65] Perez DL , Nicholson TR , Asadi-Pooya AA , Bègue I , Butler M , Carson AJ , David AS , Deeley Q , Diez I , Edwards MJ and Espay AJ (2021) Neuroimaging in functional neurological disorder: state of the field and research agenda. NeuroImage: Clinical 30, 102623. 34215138 10.1016/j.nicl.2021.102623 PMC8111317

[ref66] Polito V and Liknaitzky P (2022) The emerging science of microdosing: A systematic review of research on low dose psychedelics (1955-2021) and recommendations for the field. Neuroscience and Biobehavioral Reviews 139, 104706. 35609684 10.1016/j.neubiorev.2022.104706

[ref67] Raynor G and Baslet G (2021) A historical review of functional neurological disorder and comparison to contemporary models. Epilepsy and Behavior Reports 16, 100489. 34755104 10.1016/j.ebr.2021.100489 PMC8564048

[ref68] Remigio-Baker RA , Hungerford LD , Bailie JM , Ivins BJ , Lopez J and Ettenhofer ML (2024) The patient global impression of change as a complementary tool to account for neurobehavioral and mental health symptom improvement for patients with concussion. Disability and Rehabilitation 47(1), 235–243. 38821113 10.1080/09638288.2024.2346233

[ref69] Ricciardi L , Demartini B , Crucianelli L , Krahé C , Edwards MJ and Fotopoulou A (2016) Interoceptive awareness in patients with functional neurological symptoms. Biological Psychology 113, 68–74. 26528552 10.1016/j.biopsycho.2015.10.009

[ref70] Sharot T , Korn CW and Dolan RJ (2011) How unrealistic optimism is maintained in the face of reality. Nature Neuroscience 14, 1475–1479. 21983684 10.1038/nn.2949 PMC3204264

[ref71] Simonsson O , Johnson MW and Hendricks PS (2024) Psychedelic and MDMA-related adverse effects-a call for action. JAMA Health Forum 5(11), e243630. 39514192 10.1001/jamahealthforum.2024.3630

[ref72] Snyder ES , Tao P , Svetnik V , Lines C and Herring WJ (2021) Use of the single-item patient global impression-severity scale as a self-reported assessment of insomnia severity. *Journal of Sleep Research* 30. 10.1111/jsr.13141 33210445

[ref73] Sojka P , Diez I , Bareš M and Perez DL (2021) Individual differences in interoceptive accuracy and prediction error in motor functional neurological disorders: A DTI study. Human Brain Mapping 45, 1434–1445. 10.1002/hbm.25304 PMC7927304 33615622

[ref74] Soto CJ and John OP (2017) The next Big Five Inventory (BFI-2): developing and assessing a hierarchical model with 15 facets to enhance bandwidth, fidelity, and predictive power. Journal of Personality and Social Psychology 113, 117–143. 27055049 10.1037/pspp0000096

[ref75] Spitzer RL , Kroenke K , Williams JB and Löwe B (2006) A brief measure for assessing generalized anxiety disorder: the GAD-7. American Medical Association 166, 1092–1097. 10.1001/archinte.166.10.1092 16717171

[ref76] Stone J , Warlow C and Sharpe M (2010) The symptom of functional weakness: a controlled study of 107 patients. Brain 133, 1537–1551. 20395262 10.1093/brain/awq068

[ref77] Studerus E , Kometer M , Hasler F and Vollenweider FX (2011) Acute, subacute and long-term subjective effects of psilocybin in healthy humans: a pooled analysis of experimental studies. Journal of Psychopharmacology 25, 1434–1452. 20855349 10.1177/0269881110382466

[ref78] Tap SC , Thomas K , Páleníček T , Stenbæk DS , Oliveira-Maia AJ , van Dalfsen J and Schoevers R (2025) Concomitant use of antidepressants and classic psychedelics: a scoping review. Journal of Psychopharmacology 39(10), 1072–1088. 40937732 10.1177/02698811251368360 PMC12572353

[ref79] Ware JE , Snow KK , Kosinski M and Gandek B (1993) SF-36 health survey: manual and interpretation guide. Boston, MA, USA: The Health Institute, England Medical Center.

[ref80] Watts R and Luoma JB (2020) The use of the psychological flexibility model to support psychedelic assisted therapy. Journal of Contextual Behavioral Science 15, 92–102.

[ref81] Weiss F , Magnesa A , Gambini M , Gurrieri R , Annuzzi E , Elefante C , Perugi G and Marazziti D (2025) Psychedelic-induced neural plasticity: a comprehensive review and a discussion of clinical implications. Brain Sciences 15(2), 117. 40002450 10.3390/brainsci15020117 PMC11853016

[ref82] Wilde A (2023) The current research on psychedelic therapy for chronic fatigue syndrome (CFS), Available at: https://www.psychedelicpassage.com/the-current-research-on-psychedelic-therapy-for-chronic-fatigue-syndrome-cfs/.

[ref83] Yang Y , Wang Y and Wang X (2025) Harnessing psychedelics for stroke recovery: therapeutic potential and mechanisms. Brain 148(6), 1862–1865. 40043182 10.1093/brain/awaf093

[ref84] Yerubandi A , Thomas JE , Bhuiya NMMA , Harrington C , Villa Zapata L and Caballero J (2024) Acute adverse effects of therapeutic doses of psilocybin: a systematic review and meta-analysis. JAMA Network Open 7, E245960. 38598236 10.1001/jamanetworkopen.2024.5960 PMC11007582

[ref85] Younger J , Gandhi V , Hubbard E and Mackey S (2012) Development of the stanford expectations of treatment scale (SETS): a tool for measuring patient outcome expectancy in clinical trials. Clinical Trials 9: 767–776. 23169874 10.1177/1740774512465064

[ref86] Yozbatiran N , Der-Yeghiaian L and Cramer SC (2008) A standardized approach to performing the action research arm test. Neurorehabilitation and Neural Repair 22, 78–90. 17704352 10.1177/1545968307305353

